# Mechanisms of Disease in Sjögren Syndrome—New Developments and Directions

**DOI:** 10.3390/ijms21020650

**Published:** 2020-01-19

**Authors:** Cintia S. de Paiva, Stephen C. Pflugfelder

**Affiliations:** Department of Ophthalmology, Baylor College of Medicine, Houston, TX 77030, USA; stevenp@bcm.edu

## 1. Introduction

Sjögren Syndrome (SS) is an autoimmune disease that affects the exocrine glands, mainly salivary and lacrimal glands. Clinically, patients often complain of fatigue, dry mouth, and dry eye. This special issue about “Mechanisms of Disease in Sjögren Syndrome” publishes a total of 17 peer-reviewed articles (five reviews and 12 original studies). The call for papers did not specify any specific subject, but several major themes were observed and are described below.

## 2. Type 1 and Type 2 Interferons in SS

One theme of this special issue is the crucial role that Type 1 and 2 interferons and their inducible factors play in the pathogenesis of mucosal and glandular diseases in patients and representative animal models of SS. 

### 2.1. Interferons and Clinical Disease

Cathepsin S is an IFN-γ inducible gene [1], and increased tear cathepsin S activity has been reported in SS [2]. Klinngam et al. reports that treatment of cultured human corneal epithelial cells with recombinant human cathepsin S for 2–24 hours increased expression of interleukin 1 (IL-1), IL-6, IL-8 and TNF-α after 2–4 hours, and matrix metalloproteinase 9 (MMP-9), cathepsin S and protease-activated receptor 2 after 24 hours [3]. Silencing of protease-activated receptor 2 expression reduced the inflammation stimulating effects of cathepsin S, indicating that the effects of cathepsin S are mediated by protease-activated receptor 2 activation. 

Increased acinar epithelial cell death contributes to the secretory dysfunction of exocrine glands in SS. Nakamura et al. review the pro- and anti-apoptotic pathways that impact salivary gland acinar cell survival [4]. Apoptosis is promoted by FAS-FASL, TRAIL, and TLR3 signaling and is suppressed by the pro-survival factors EGF and X-chromosome-linked inhibitor of apoptosis. TLR signaling has been found to increase the production of caspase 3 and pro-apoptotic cytokines.

Pflugfelder and colleagues report an increased number of HLA-DR^+^ antigen-presenting and CD11c^+^CD86^+^ dendritic cells in SS bulbar conjunctiva compared to control non-dry eyes [5]. The percentage of HLA-DR^+^ cells positively correlated with clinical severity APCs. Both HLA-DR and CD86 are IFN-γ inducible genes.

Reis de Oliveira et al. review the evidence that the Kynurenine metabolic pathway that is activated by IFN-γ can interfere with serotonergic and glutamatergic neurotransmission in the CNS [6] and contribute to the hyperalgesia, depression and disconnect between clinical signs and symptoms that are frequently observed in SS. 

### 2.2. Animal Models

Chaly et al. study the expression of genes that are associated with the development of dacryoadenitis in SS-prone NOD and MRL/lpr mouse strains [7] with increased expression of 5 candidate genes with potential roles in mechanisms upstream of lacrimal gland lymphocytic infiltration. Among these, three are regulated by Type 1 IFN and disease was prevented in NOD/Type 1 IFN receptor (Ifnar1) deficient mice. 

Knox et al. evaluate the evolution of dacryoadenitis in the AIRE knockout mouse that develops SS-like disease [8]. They report significantly increased expression of inflammatory mediators, including IFN-γ, IL-1β, NFκB and toll-like receptor signaling molecules in the lacrimal glands of autoimmune response element (AIRE) knockout mice that develop SS-like at five weeks of age, two weeks prior to the onset of severe lymphocytic infiltration and decreased secretory function of the glands, indicating these factors may be valuable diagnostic and severity biomarkers. 

Ogawa et al. review the evidence that IFNs contribute to the early innate and later lymphocyte-mediated stages of SS [9]. Innate APCs produce IFN-α and -γ, while NK cells produce IFN-γ. Both Type 1 and 2 interferons stimulate BAFF production by glandular epithelia which in turn stimulates B cell activation and their maturation to autoantibody-producing plasma cells. IFN-γ, also produced by Th1 effector cells, promotes apoptosis of glandular and mucosal epithelia and induces endoplasmic reticulum stress and unfolded protein response in goblet cells, resulting in reduced mucin secretion.

Collectively, these studies present strong evidence that IFN signaling has critical disease-promoting activity in SS, promoting exocrine gland dysfunction, keratoconjunctivitis sicca, and neurological/psychological manifestations that decrease quality of life. They indicate that IFN signature molecules could serve as disease biomarkers and provide a rationale for the development of therapies that target these factors. 

## 3. Novel Biomarker

Epidermal fatty-acid binding protein (E-FABP or FABP5) is an intracellular chaperone molecule involved in the transport of long-chain unsaturated fatty acids. Shinzawa and colleagues show a significant decrease in the E-FABP protein concentration in tears of patients with SS compared to healthy controls. Interestingly, there were no between-group differences in saliva or serum. Furthermore, E-FABP protein levels correlated with dry eye parameters, suggesting that E-FABP levels could be used as a biomarker for dry eye [10].

## 4. Environmental Influences in SS

SS is a multifactorial disease that is not yet completed understood. Although some polymorphisms in HLA-DR and TNF-α have been identified [11], the concordance in monozygotic twins is low [12]. Environmental factors are yet to be fully identified. Three research articles shed light on environmental clues triggering/worsening SS.

Wang and colleagues demonstrate that germ-free C57BL/6 spontaneously develop an SS-like syndrome [13]. They observed that corneal barrier disruption (or corneal epitheliopathy) and lacrimal gland infiltration and gene expression were significantly altered in females compared to males, while conjunctival goblet cell loss was similar in both sexes. They also identified a greater percentage of IL-12 producing antigen-presenting cells in the conjunctiva and draining cervical lymph nodes. This was accompanied by increased CD4^+^IFN-γ^+^ cells in lacrimal glands and adoptive transfer of germ-free CD4^+^ T cells recapitulated disease in immunodeficient hosts. Colonization of germ-free mice with fecal intestinal microbiota reversed the dry eye phenotype and decreased the pathogenicity of CD4^+^ T cells. Their results indicate that commensal bacteria have a protective role in SS.

One frequent finding in dysbiosis (or microbial imbalance) is the relative increase in proteobacteria, such as Shigella/Escherichia, which in baseline/homeostasis constitute less than 1% of intestinal bacterial communities [14]. Yanagisawa and colleagues show that mice repeatedly treated with IP injections of *E. coli* developed salivary and Harderian gland infiltration after 8 weeks [15]. They further identified a component of the outer membrane of *E. coli* as sufficient to induce extra-intestinal inflammation in salivary, Harderian glands, but not in the pancreas. This was accompanied by increased levels of SS-A and SS-B antibodies. The lacrimal gland was not investigated. Their results point out a pathogenic role of OMP-A, an outer membrane protein of *E. coli*. 

Mircheff et al. investigate the expression of cytokines and chemokines in lacrimal glands in healthy nulliparous and multiparous rabbits of different ages [16]. Using principal component analysis, they identified several clusters of genes associated with SS foci. C–X–C motif chemokine ligand 13 (CXCL13) and B-cell activating factor (BAFF) dominated the major component. Using laser capture dissections, they also identified clusters of genes associated with acinar cells, ducts, and immune cell aggregates. Pregnancy and dryness influenced the results.

## 5. B Cells and SS

One of the features of SS is B cell infiltration of the exocrine glands and production of autoantibodies, including, but not limited to, Ro/SSA, SSB, and muscarinic 3 receptor [17,18]. Trzeciak and colleagues show that immunization of NZM2758 mice with recombinant Ro52 protein, an antigen recognized by SSA antibodies, induced decreased tear volume, increased immune infiltrates, and IgG deposits in the lacrimal gland [19]. Interestingly, male mice were protected and these changes were only observed in female mice, suggesting that females are more susceptible to immune-mediated damage.

Singh and colleagues show that in male Thrombospondin 1^−/−^ mice, a spontaneous SS model, there is an increased number of marginal zone B cells in the spleens, lacrimal glands and conjunctiva compared to wild-type mice [20]. Furthermore, immunization of these mice with ovalbumin (OVA), followed by administration of a thrombospondin specific peptide (TSP-derived peptide N1K), decreased the levels of OVA-specific IgG and decreased marginal zone B cell dysregulation in the spleen.

## 6. Corneal Nerves

There is a well-documented disconnection between dry eye symptoms and signs in dry eye [21]. Severe dry eye patients often do not complain of irritation to the same level as the severity of corneal epitheliopathy. One of the reasons is reduced corneal nerve density [22]. Two articles examine corneal nerves in two different animal models of SS. 

Tatematsu et al. investigate the structure of corneal nerves, specifically neurotransmitter containing nerves in female Thrombospondin 1^−/−^ mice of two different ages (4–7 and 9–12 weeks) [23]. Compared to age-matched wild-type mice, Thrombospondin 1^−/−^ mice had a lower number of corneal nerves both by immunofluorescence in wholemount specimens and in vivo confocal microscopy, which worsened by 12 weeks. The most significant decrease was in calcitonin gene-related peptide-containing nerves, but not in Substance P nerves. Significantly elevated levels of monocyte chemoattractant protein-1 (MCP-1), macrophage inflammatory protein-2 (MIP-2), and TNF-α were detected in corneas of 12-week old Thrombospondin 1^−/−^ mice.

The CD25 null mouse is a well-established animal model of SS, with ocular and lacrimal gland involvement. Stepp and colleagues show decreased axon density, stromal arborization, and increased Ki67+ cells in corneas of CD25 null mice compared to age-matched wild-type littermates [24]. They also observed decreased corneal mechanical sensitivity and increased mRNA levels of genes involved in phagocytosis and autophagy (including Beclin1, LC3, LAMP1, LAMP2, and brain-derived neurotrophic factor (BDNF)).

## 7. Reviews

Two reviews describe exocytosis mechanisms and the role of aquaporins in SS.

Suzuki and Iwata review the current molecular knowledge of exocytosis and glandular secretion in both salivary and lacrimal glands. A detailed overview of the exocytosis process, as well as descriptions of SNARE (soluble N-ethylmaleimide-sensitive factor attachment protein (SNAP) receptor) proteins, are provided in SS and homeostasis [25].

Aquaporins are a family of water-permeable channels. Soyfoo and colleagues review the role of aquaporins in the diagnosis and treatment in SS, with a focus on the salivary gland alterations that accompany SS. The authors discuss how alterations in distribution, localization and the presence of autoantibodies to aquaporin 5 might have a role promoting SS [26].
